# Eleven Letters with Answers

**Published:** 1878-04

**Authors:** 


### Our Correspondence.

PETROLIA, PA., Jan. 10, 1878.

THAD S. UP DE GRAFF, M. D.:

Enclosed please find your blank filled and 50 cents for one years subscription to your lively little quarterly. Shall show the number I now have to my medical friends, and probably shall succeed in getting you quite a number of subscribers here in the oil country. I like the little journal on account of its practical teachings.

Respectfully,
L. M. W., M. D.

Wish more of our patrons would follow Dr. W.'s example, and send us extra subscribers. If they will only exhibit the BISTOURY to their friends, it will recommend itself on sight. Our journal is capable of doing an immense amount of good in every family, in the simple matter of cautioning the members thereof against the impositions of quacks that infest almost every city.

---

ONAWA, IOWA, Jan. 14, 1878.

DR. UP DE GRAFF:

*Dear Sir*:—In your January number of the BISTOURY, I notice an article in your correspondence column, about Ayers' Hair Vigor, and your answer. Now, I am an old school physician of over twenty years practice, and I take your BISTOURY because I like the way you handle quacks. I never gave, nor recommended a patent medicine in my life, but I will give for the benefit of your readers, a prescription of my own, which has grown me a good head of hair, where I was quite bald one year ago, and it will not do any injury either.

℞

Ol ricini, ℥j.
Alcohol.
Tr. lobelia, āā ℥ iv.

M. Use a little once or twice a day, rubbing with a stiff brush. Any perfume may be added.

J. BUTTS, M. D.

---

HISEVILLE, KY., Jan. 22, 1878.

THAD S. UP DE GRAFF, M. D.:

*Dear Sir*:—Your most welcome and excellent journal has become a household fixture and necessity. I could not do without it without feeling the loss of it greatly. It has been my constant companion for ten years or more.

Hoping for you a prosperous, happy new year, and that you may still live many long years of prosperity and usefulness,

I am, sincerely your friend,
S. L. N., M. D.

We give this as a sample of bushels of letters received, expressing admiration for the BISTOURY.

---

HAMMONDSPORT, N. Y., Feb. 18, 1878.

DR. UP DE GRAFF, Elmira, N. Y.:

*Dear Sir*:—I take the liberty of writing you in reference to the manufacture and application of false parts to the face. That is, do you make or apply to persons, say, a false nose? If so, you will confer a favor by informing me the cost of the same. The necessary formula to go through, and if it is necessary for the person to visit you. And if not doing this kind of work, can you inform me where it is done?

Yours respectfully, E. B. L.

Artificial noses are made of silver, enameled so as to take the hue of the skin, and then held in position by means of spectacle frames. They look very well, and hide an otherwise hideous deformity, where the nose is lost from disease. Much the best plan, however, is to apply to some skillful surgeon and have a nose healed on your face, made of the integument of the forehead or of the arm. This is frequently done, and makes a very satisfactory looking nose, not liable to drop off, or bob about in exercising, and can be grasped and blown with some degree of satisfaction.

---

TROY, ALA., Feb. 21, 1878.

DR. UP DE GRAFF:

Where can Ringer's Hand Book of Therapeutics, mentioned in the last number of the BISTOURY, be obtained, and the price? J. D., M. D.

Of Wm. Wood & Co., 27 Great Jones Street, New York. Price, $4.50.

As we have had a great number of inquiries for this book, the above will answer for all. In writing for it, please mention that you saw the notice in the BISTOURY.

---

TOWANDA, PA., Feb., 1878.

DR. UP DE GPAFF:

*Dear Sir*:—Will you please send me a receipt for removing superfluous hair. Do you think the following a good and safe receipt: With two parts of quick-lime, mix one of sulphuret of arsenic, and one of starch. I enclose stamp. Please answer by return mail.

Yours respectfully, D.

The receipt is as good as any. There are no depilatories that are entirely satisfactory. The best plan is to destroy one hair bulb at a time, by means of pure nitric acid, applied to the root of the hair, upon the point of a sharp pine stick. The stick is dipped into the acid, all excess shaken off, and its sharp point pushed down to the bulb by the side of the hair.

---

JACKSONVILLE, FLA., Feb. 27, 1878.

THAD S. UP DE GRAFF, M. D.:

*Sir*:—As I did not receive the January number of the BISTOURY, I fear my subscription has run out, so I enclose one dollar to renew it for two years.

I feel that I cannot be without the BISTOURY, and look for the quarter to come around when it will put in an appearance. I wish the keen edge of your BISTOURY might be felt by some of the gorilla M. D.'s who practice somewhere in the north during the summer, then follow the invalids to this more pleasant climate and palm themselves off with a long string of references, an equal one of the honorable positions they have filled, in order to catch the poor invalids in search of health, and make merchandize of them. They take no share of the duties the profession feels towards the more unfortunate of our race, pay no license, or tax, but collect with unsparing hand of those they make their victims, and next winter seek some other fields for their prey.

Yours, truly, A. J. W., M. D.

The Doctor, in common with his professional brethren who reside permanently at the south, have just reason of complaint of the growing evil of which he writes. There exists a class of physicians who are not worthy of the name—a lot of men who seek *only* to make money out of their profession, and who would not consent to call upon a sick person without first ascertaining whether his fee would be secure. It is this class who migrate to the southern resorts in the winter, [following the tide of invalids who seek sunshine and better health away from their northern homes], and hang out their shingles to catch the patronage of the wealthy and ailing sojourners. But, as the doctor writes, they are never found among the sick poor of the places where they have taken their temporary residences—not they, for fees do not there abound.

We know of no remedy for this injustice done the southern physician, save that which might be regulated by local laws.

---

ELMIRA, N. Y., March 1, 1878.

DR. UP DE GRAFF :

I have catarrh. I wrote to the man whose circular I enclose, for a free formula to cure my disease. He sent me this prescription, and the druggists have not got all the ingredients. Is it all right? M.

The circular referred to is from "Rev. Warren R. Williams, D. D.," of Syracuse, N. Y., who recommends the applicant, in answer to his advertisement, to send $5 to "A. Humbolt Barrow, M. D.," of the same city, for the prescription already prepared, in case it cannot be prepared by the druggist. As no druggist *can* prepare the formula—it containing the names of drugs known only to the fertile brain of the advertiser—of course, many idiots would patronize the swindle. It is always safe to say you can never get *something* for *nothing.* No man is likely to go to the expense of advertising a cure for catarrh, or any other disease, and take pay in "gratitude"—not much. This system of swindling would seem too "thin" even to catch a booby, but it does now and then.

---

NEW YORK, March 1, 1878.

DR. UP DE GRAFF :

I enclose you a circular which I sent for. As the artificial ear drums only cost three dollars, I thought I would order a pair. Will they do me any good? G. S.

Not a particle. Better let John Garmore and his ear drums alone. They are nothing new under the sun. Ear drums are inserted by Aurists where the natural drum has been carried away through disease, (*otorrhœa*, usually), and are *never* used under any other circumstances. To apply them in a case of "nervous deafness," like yours, is worse than useless.

John is either a fraud, else is he not well versed in aural diseases. Likely both.

---

BINGHAMTON, N. Y., Mar. 28, 1878.

DR. UP DE GRAFF :

*Dear Sir:* * * * * * That Bistoury of yours is one of the sharpest of instruments. I never read a journal that was so thoroughly chuck full of good things. Why in creation do n't you make it a monthly? See here! I will agree to send you fifty subscribers from this city, at a dollar each, if you will do it! I get hungry for the Bistoury long before the quarter expires. * * * * What has become of the Great English Physician that you flailed so unmercifully two years ago? He promised to return here, but the Bistoury exposure, doubtless, changed his designs. W.

We agree perfectly with our correspondent, relative to the value of our journal. Sorry we cannot, now, make it a monthly. Such a procedure would entail more labor upon us than we can give. *Some day* we hope to see the Bistoury a monthly.

The Doctor (?) referred to, is in trouble again, as will be seen in our local.

---

A gentleman writes us from Blossburg, Pa., enquiring what can be done with an eye that was made blind with "Thompson's Eye Water."—Do n't know. Anybody foolish enough to use patent medicine in an inflamed eye, we had almost said, deserves to lose it.

